# Efficient isolation on Vero.DogSLAMtag cells and full genome characterization of Dolphin Morbillivirus (DMV) by next generation sequencing

**DOI:** 10.1038/s41598-018-19269-2

**Published:** 2018-01-16

**Authors:** Simone Peletto, Claudio Caruso, Francesco Cerutti, Paola Modesto, Cristina Biolatti, Alessandra Pautasso, Carla Grattarola, Federica Giorda, Sandro Mazzariol, Walter Mignone, Loretta Masoero, Cristina Casalone, Pier Luigi Acutis

**Affiliations:** 1 Istituto Zooprofilattico Sperimentale del Piemonte, Liguria e Valle d’Aosta, Turin, Italy; 2 Department of Comparative Biomedicine and Food Science, University of Padua, Padua, Italy

## Abstract

The Dolphin Morbillivirus (DMV) genome from the first Mediterranean epidemic (1990-’92) is the only cetacean *Morbillivirus* that has been completely sequenced. Here, we report the first application of next generation sequencing (NGS) to morbillivirus infection of aquatic mammals. A viral isolate, representative of the 2006-’08 Mediterranean epidemic (DMV_IZSPLV_2008), efficiently grew on Vero.DogSLAMtag cells and was submitted to whole genome characterization by NGS. The final genome length was 15,673 nucleotides, covering 99.82% of the DMV reference genome. Comparison of DMV_IZSPLV_2008 and 1990-’92 DMV strain sequences revealed 157 nucleotide mutations and 47 amino acid changes. The sequence similarity was 98.7% at the full genome level. Whole-genome phylogeny suggested that the DMV strain circulating during the 2006-’08 epidemics emerged from the 1990-’92 DMV strain. Viral isolation is considered the “gold standard” for morbillivirus diagnostics but efficient propagation of infectious virus is difficult to achieve. The successful cell replication of this strain allowed performing NGS directly from the viral RNA, without prior PCR amplification. We therefore provide to the scientific community a second DMV genome, representative of another major outbreak. Interestingly, genome comparison revealed that the neglected L gene encompasses 74% of the genetic diversity and might serve as “hypervariable” target for strain characterization.

## Introduction

Cetacean morbillivirus (CeMV) is a member of the genus *Morbillivirus*, which includes several virus species with a single-stranded negative-sense RNA genome. Morbilliviruses affect mammals causing measles in humans, peste des-petits-ruminants in ruminants, rinderpest in cattle, phocine distemper in seals, and canine distemper in carnivores^1,2^.

Three strains of CeMV, namely dolphin morbillivirus (DMV), porpoise morbillivirus (PMV) and the pilot whale morbillivirus (PWMV), previously considered as distinct viruses, are now classified as one species and constitute the “old” CeMV lineage, which also includes the recently discovered beaked whale morbillivirus (BWMV)^3^. Other two new strains of morbillivirus have been recently detected, in Indo-Pacific Bottlenose dolphin and in Guiana dolphin^4,5^, representing the “new” CeMV lineage^3^.

Nowadays, CeMV is regarded as an emerging threat being the cause of recent epidemics worldwide^6^. In 1990-’92, a DMV outbreak in the Mediterranean Sea involved thousands of striped dolphins (*Stenella coeruleoalba*) with high mortality^7^. Another major epidemic event was the DMV outbreak in 2006–2008 affecting three cetacean species along the Mediterranean coast: the striped dolphin *S. coeruleoalba*, the bottlenose dolphin *T. truncatus* and the long-finned pilot whale *Globicephala melas*^8–11^.

The DMV genome from the 1990-’92 epidemic is the only CeMV genome that has been completely sequenced^12^. Similarly to other Morbillivirus members, the DMV genome was determined to be a non-segmented, negative single-stranded, RNA molecule about 15.7 kb long and containing six genes; these genes encode eight proteins, six of which are structural, and they are organized in the following order: 3′ nucleoprotein (N)–phosphoprotein (P)–matrix protein (M)–fusion protein (F)–hemagglutinin protein (H)–large protein (L) 5′^12^.

From a diagnostic point of view, isolation of DMV on cell cultures is still regarded as the “gold standard” for definitive diagnosis of infection, though it remains a challenge when attempted from stranded cetaceans^3^. Actually, most of what is known about Morbillivirus infections and epidemiology in marine mammals has come from molecular assays (RT-PCR), since wild-type strains are difficult to isolate and propagate in cell culture systems^3,13^. Previous studies indicated that African Green Monkey Kidney (Vero) cell line may be suitable for DMV isolation; more recently, a new developed Vero cell line expressing the Canine Signaling Lymphocyte Activation Molecule (SLAM/CD150) showed to be permissive for DMV replication^13^.

In this study, we report the first retrospective isolation of DMV from a striped dolphin (*Stenella coeruleoalba*) stranded along the Ligurian coast of Italy in 2008, within the Pelagos Sanctuary (http://www.sanctuaire-pelagos.org/en/). This viral isolate, representative of the 2006-’08 Mediterranean epidemic, efficiently grew on Vero.DogSLAMtag cells and was submitted to whole genome sequencing by next generation sequencing (NGS) technology. We therefore provide the second genome sequence of a cetacean morbillivirus; the sequence of the DMV is near complete and a genome comparison with the 1990-’92 DMV strain was carried out.

## Results

**Figure 1:**
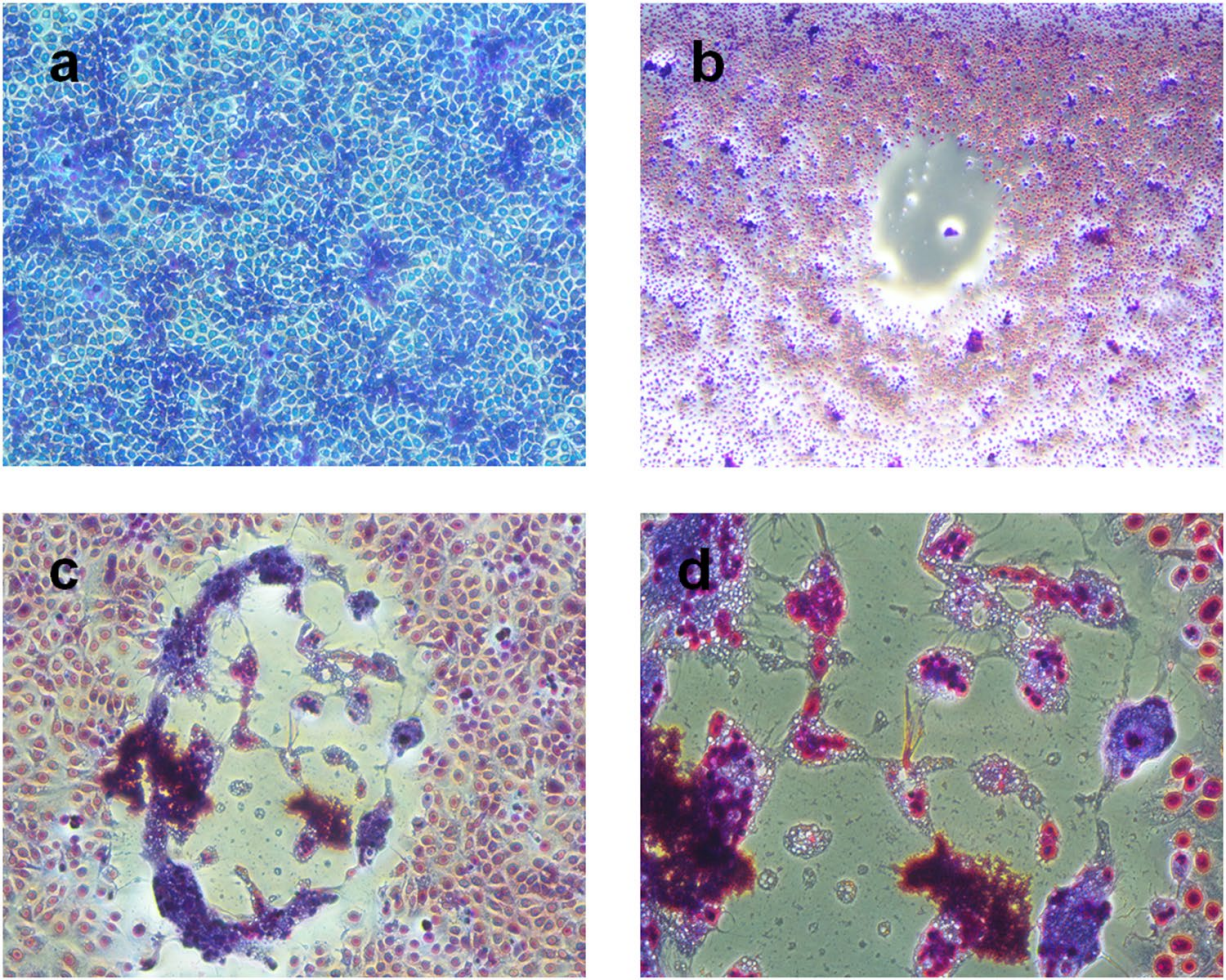
(**a**) Vero/dog SLAM, mock infected cells. 10X, 48 hours; (**b**) Vero/dog SLAM, syncitium. 4X, 4–5 days post infection (p.i.); (**c**) Vero/dog SLAM, syncitium and round-up of cells; 10X, 4–5 days p.i.; (**d**) Vero/dog SLAM, syncitium and round up of cells. 20X; 4–5 days p.i.

Successful viral isolation on Vero.DogSLAMtag cell line was demonstrated by CPE observed after five days (first passage), consisting in syncitia formation and rounding-up of cells. The plaques showed up as clear areas in the Giemsa-stained cell monolayers (Fig. 1). DMV propagation was then confirmed by RT-PCR positivity in the supernatant.

The sequencing run delivered 4.1 Gb of sequence data corresponding to a total of 12,026,922 generated reads. Adapters and low-quality bases were trimmed from the sequence reads and the remaining reads were 12,856,456. In total, 3,196 reads of DMV were acquired and mapped to the DMV reference genome (GenBank Acc. no. NC_005283.1) with a normalized average coverage of 29.3×. De novo assembly using CLC Genomics Workbench v. 10.1.1 resulted in six DMV contigs, with a contig length ranging from 366 to 4,528 nucleotides (nts). In parallel, reads were de novo assembled using SPAdes v 3.10.0 with the default settings, generating a single DMV contig of 15,693 nts, which was manually trimmed at the 3′ end. The final length obtained from the read assembly was 15,673 nts covering 99.82% of the DMV reference genome obtained from a striped dolphin infected during the 1990-’92 epidemics. The alignment between the consensus generated for the studied isolate (hereafter named DMV_IZSPLV_2008) and the reference sequence showed that the genomic regions not covered by sequence reads corresponds to 27 and 3 putative nts of the viral RNA ends (5′ leader and 3′ trailer, respectively).

The genome of the DMV_IZSPLV_2008 isolate contains six non-overlapping genes, in the order N/P -V - C/M/F/H/L, typical of Morbillivirus, coding for eight structural and non-structural proteins. The amino acid (aa) length of the six structural proteins encoded by the DMV_IZSPLV_2008 genome is: N, 523 aa; P, 506 aa; M, 335 aa; F, 552 aa; H, 604 aa; L, 2183 aa.

**Figure 2:**
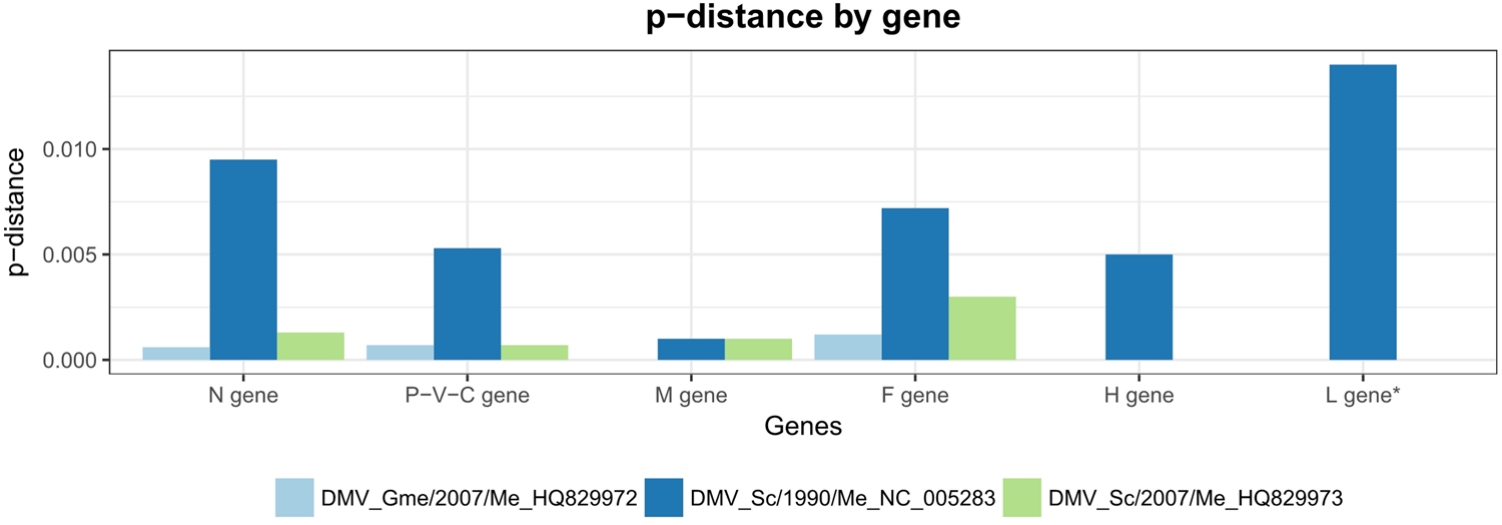
Mean nucleotide p-distance by gene between the DMV_IZSPLV_2008 isolate and available DMV genomic sequences representative of the 1990-’92 and 2006-’08 outbreaks in the Mediterranean Sea. (*) Comparison of the L genes was carried out only between the 1990-’92 DMV and DMV_IZSPLV_2008 isolates for which sequences of this region are available.

The sequence similarity between the DMV_IZSPLV_2008 genome and the reference, representative of the 1990-’92 epidemics in the Mediterranean Sea, was 98.7% at the nucleotide level. Sequence alignment comparing the two genomes revealed mutations at 157 nucleotide sites. Additionally, the DMV_IZSPLV_2008 isolate was compared with two partial sequences identified in Spain during the 2006-’08 outbreak from a striped dolphin and a pilot whale (GenBank Acc.nos HQ829973 and HQ829972, respectively) restricting the analyzed genomic region to fit their length (9,050 nt, ranging from the 3′ end to the complete CDS of the H gene). In this case, sequence similarities were 99.8% and 99.9%, respectively. The similarity relative to this partial genomic region with the reference genome was 99.3% corresponding to 64 nucleotide changes. The nucleotide diversity (p-distance) across genes of the above mentioned DMV genomic sequences ranged from 0.000 to 0.014 (Fig. 2).

**Figure 3:**
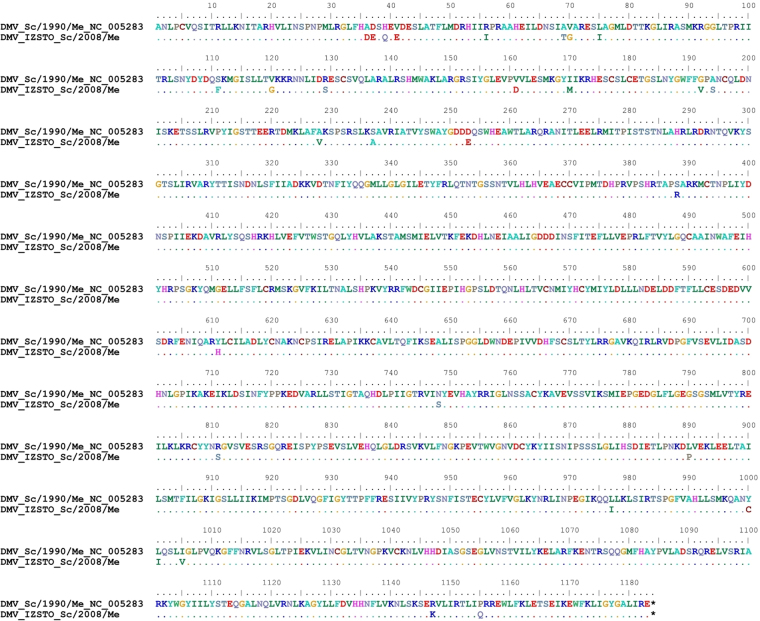
Large protein (L) sequences of the DMV reference genomes representative of the 1990-’92 and 2006-’08 outbreaks in the Mediterranean Sea. Points indicate identical residue. Host species (Sc = *Stenella coeruleoalba*), collection date and accession numbers are indicated. The sequence marked by asterisk (*) has been newly determined in this study.

Several aa changes compared to the DMV reference genome have been identified, specifically: 4 aa in the N protein, 3 aa in the P protein, 1 aa in the M protein, 3 aa in the F protein, 1 aa in the H protein (Supplementary Figures S1-S5) and 35 aa in the L protein (Fig. 3). To confirm novel (i.e. not reported in the reference DMV genome) amino changes, PCR amplification of short genomic regions was carried out using as a template total RNA isolated directly from the dolphin brain. All coding nucleotide variations identified by NGS were confirmed by Sanger sequencing. At the amino acid level, identities between the viral proteins of DMV_IZSPLV_2008 and the reference 1990-’92 isolate were: 99.2% (N protein), 99.4% (P protein), 99.7% (M protein), 99.4% (F protein), 99.8% (H protein), and 98.4% (L protein). The same comparison carried out between the Italian isolate and the two partial sequences from Spain showed 99.6% similarity at both N and F proteins, and 100% identity at P, M and H proteins.

**Figure 4:**
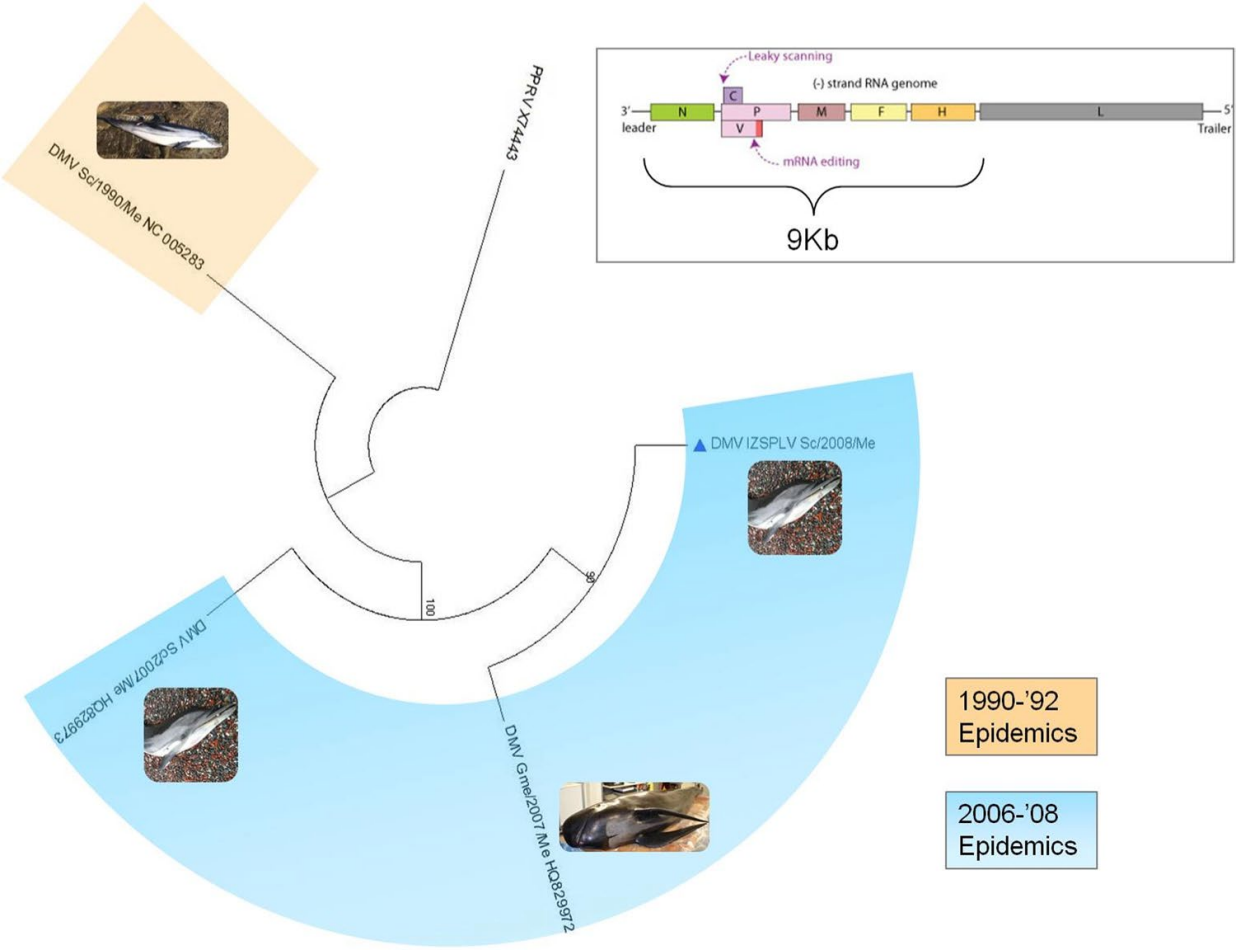
MEGA7: phylogenetic tree constructed by Maximum Likelihood (ML) analysis from an alignment of 9,039 nt, corresponding to the complete N, P, M, F, H genes and a 5′ fragment of the L gene (first 10 residues). The tree includes the DMV_IZSPLV isolate (identified by a blue triangle) and sequences available in GenBank. Host species (Sc = *Stenella coeruleoalba*; Gme = *Globicefala melas*), collection date and accession numbers are indicated for each sequence. Bootstrap values (1,000 iterations) >70 are shown at the internal nodes. The tree is drawn to scale, with branch lengths measured in the number of substitutions per site.

Figure 4 shows the phylogenetic tree obtained using the maximum likelihood method (ML), which includes the above mentioned sequences and the Peste-des-petits-ruminants virus (PPRV) genome as an outgroup.

## Discussion and Conclusions

In Morbillivirus diagnostics, viral isolation is still considered the “gold standard”, even though efficient *in vitro* isolation and propagation of live virus is difficult to achieve. As underlined by Nielsen *et al*.^13^, the identification of the SLAM/CD150 as universal cell receptor molecule for morbillivirus attachment and the use of stably transfected cells expressing these receptors offer a substantial improvement over traditional cell culture methodologies for isolation and characterization of marine mammal morbilliviruses. In our study, Vero.DogSLAMtag cells allowed the isolation of a wild strain of DMV from an infected striped dolphin brain after 8-years of storage in the tissue bank of C.Re.Di.Ma (Italian National Reference Centre for Diagnostic Investigations on Stranded Marine Mammals). This result seems to confirm the high sensitivity of this cell line for isolating cetacean morbillivirus; importantly, extensive CPE was evident after only 48 h of incubation. Remarkably, virus isolation was also attempted using other cell lines (Vero and Vero h/SLAM). However, no viral replication could be appreciated, as assessed by the lack of CPE, positive staining of the cell monolayer, and absence of DMV nucleic acids through passages. With the development of non-cultural methods for the rapid detection of pathogens, the usefulness of viral isolation assays has been questioned; however, this is not true for cetacean morbillivirus since the recovery and isolation of infectious virus has represented a limit to the diagnostics, characterization and complete epidemiology understanding of epidemic strains so far. Availability of infectious virus is the first step to obtain polyclonal and monoclonal antibodies for confirmatory testing of virus cultures showing CPE, traditionally based on the reaction of antibodies of known specificity with viral antigens expressed in the infected cells^14^. Currently, a limitation in the indirect diagnosis of DMV infection is represented by the employment of a “heterologous” antigen (Canine Distemper Virus) for detecting anti-morbilliviral antibodies in serum neutralization assays^15^. Although this approach has been widely used in several seroepidemiological surveys, the availability of a “homologous” (DMV) antigen could result in higher sensitivity and specificity. Finally, propagation of viral isolates on cell cultures can be considered a kind of “selective enrichment method” of the pathogen towards the host background with the advantage that new approaches, like sequencing by NGS technology, can be exploited. To confirm coding variants identified by NGS, Sanger sequencing was performed on viral RNA amplified directly from the dolphin brain, thus demonstrating that *in vitro* DMV replication did not lead to the selection of minor variants or to nucleotide changes introduced during cell passages of the virus.

In our study, the successful on-culture replication of a DMV strain from an adult female of striped dolphin stranded on the Ligurian coast in 2008 allowed to perform massive parallel sequencing by NGS directly from the viral RNA without prior PCR amplification. To date, the only full genome sequence of a CeMV available in GenBank derives from a sample of striped dolphin from the 1990-’92 epidemics. In this study, we therefore provide the scientific community a second, nearly complete, CeMV genome representative of another major outbreak, that is the 2006-’08 epidemics in the Mediterranean Sea. With the availability of this sequence, we investigated whether the high level of homology among strains of the two epidemics, already observed by Bellière *et al*. (2011) but limited to partial genomic regions, would be confirmed on the entire DMV genome.

Analysis of nucleotide sequence similarity revealed an identity of 98.7% between the two genomes, thus confirming that the two viruses are highly correlated. The same analysis carried out on a partial region of 9,050 nt, to include two Spanish sequences representative of the 2006-’08 epidemics, demonstrated almost complete identity between them and the DMV_IZSPLV_2008 isolate (99.8% and 99.9%, respectively) indicating that sequences from this second outbreak belong to a unique viral strain and are more similar to each other than to the 1990-’92 DMV strain. Phylogenetic analysis performed on the same nucleotide dataset (Fig. 4) suggests that the 2006-’08 DMV strain emerged from the 1990-’92 DMV strain, in agreement with previous observations^16^. However, the limited number of available sequences requires caution in the interpretation of the tree topology at this stage. Reasonably, more full-genome sequencing is warranted.

When phylogenetic analyses were performed on each gene of DMV_IZSPLV_2008 (excluding the L gene), we found that they do not group phylogenetically with homologous genes of other CeMVs (data not shown). Indeed, they form a separate clade for each gene. This seems to confirm that homologous recombination is an uncommon event in Morbillivirus and, in general, in non-segmented negative stranded RNA viruses^17,18^. However, Park *et al*.^19^ demonstrated the existence of a naturally occurring recombinant Feline MV (FeMV) from Japan^20^. In this strain the recombination event seems to have occurred within the F and H protein encoding genes involving two very closely related parental FeMV strains.

The nucleotide diversity was determined for each gene by comparing the available genomic sequences representative of the two main Mediterranean outbreaks; p-distance values were smaller in the M gene and larger in the L gene (Fig. 2). The M gene encodes for the Matrix protein, which includes highly conserved regions and is localized on the inner side of the virus envelope^12^. Interestingly, the low level of nucleotide divergence observed at the M gene is maintained across isolates from different outbreaks, suggesting that sequence composition at this site may be less prone to be driven by evolutionary pressures. This finding would suggest the M gene as an ideal candidate region to design primers for molecular diagnostic assays. Actually, only one amino acid mutation (59 L→V) differentiates isolates from the two Mediterranean outbreaks and is located outside of a highly conserved region (Figure S3)^16,21^. On the contrary, the L gene showed the highest divergence between the only two full genome sequences: the 1990-’92 DMV and DMV_IZSPLV_2008 strains. In fact, an interesting finding is that the partial genomic region used for our phylogenetic analysis, which covers the entire CDS of the N, P, M, F, H proteins, and the first 10 amino acid residues of the L protein, although encompassing ~60% of the DMV genome, indeed includes only 26% (12 out of 47) of the diversity (amino acid changes) that differentiate the strains of the two epidemics. This is because 93 out of 157 (~59%) nucleotide mutations between the two strains are located downstream of the H gene, specifically 91 in the L gene and 2 in the 3′ untranslated trailer. These observations, despite being based only on two full genome sequences, suggest that the L protein (Large protein) gene may constitute the most variable and therefore the most informative (from a phylogenetic and molecular epidemiology point of view) region of DMV and, possibly, of CeMV in general. In fact, the comparison that we carried out on the entire genomes lowered the previously reported similarity between the 2006-’08 and 1990-’92 DMV strains from 99.3–99.4%^16^ to 98.7%, while the similarity within the three 2006-’08 DMV strains, with the inclusion of our Italian sample, was confirmed close to complete identity (99.8–99.9%). Focusing on the L protein gene, with the perspective to find internal target regions suitable for virus typing and molecular epidemiology studies, we identified a region of ~1 Kb, spanning the reference genome from positions 12,000 to 13,000, which encloses 33 out of the 91 nucleotide substitutions (96.7% strain similarity) found in the entire gene; moreover, 24 of these nucleotide substitutions were indeed located in a sub-region of ~600 bp (95,9% strain similarity), which could potentially serve as “hypervariable” target for molecular characterization of CeMV strains. The L protein, together with the phospho- (P) protein is encapsidated into a ribonucleoprotein complex (RNP) formed by the nucleocapsid (N) protein^12^. In the morbillivirus genus, the L protein harbors all enzymatic activities necessary for RNA transcription and replication^17^. From a GenBank search for CeMV sequences at the moment of writing, the only L gene sequence publicly available can be retrieved from the reference sequence of the complete 1990 DMV genome (excluding the two Spanish sequences of our phylogenetic analysis that include only a very short region corresponding to the first 10 amino acids of the L protein). Full-genome or targeted resequencing of other isolates will allow to assess the potential of the L gene, currently neglected, as a genetic target for strain discrimination, useful to gain a deeper understanding of the evolutionary relationships and of the molecular epidemiology of CeMVs.

Another interesting finding was the detection of an alanine to valine (2 A → V) mutation of the F gene located in the signal peptide sequence^21^ of the DMV_IZSPLV_2008 strain. This region is considered to be species-specific among morbilliviruses, probably because its functions are linked to those of the host cell^21,22^. Therefore, the configuration of this species-specific region in terms of amino-acid composition reflects the fact that a given morbillivirus is specific for a given host. Bellière *et al*. (2011) found that the 2 (A → V) mutation was exclusive of the DMV_Gme/2007 sequence suggesting that it might be associated to DMV adaptation to the long-finned pilot whale population. This hypothesis was also enforced by the fact that no other published morbillivirus sequences contain a valine at this position of the F protein. Despite absence of morbillivirus sequences carrying this mutation continues to hold true at the moment of writing, as assessed by a Blast search, our results seem to break the dogma of species-specificity of the N-terminal region of F protein, since the DMV_IZSPLV_2008 strain does carry the 2 (A → V) mutation. What we can speculate is that the 2 (A → V) mutation might actually have aided the virus to enter the long-finned pilot whales, however the change did not relegate DMV into this species, but probably allowed parallel circulation or reverse transmission into striped dolphins. This finding raises novel questions about the plasticity of morbilliviruses and their ability to adapt to novel host species.

In conclusion, our study is the first comparison between the complete DMV genomes obtained from the two major Mediterranean outbreaks and the first application of NGS technologies to morbillivirus infections of aquatic mammals. In the near future, this approach might facilitate obtaining a collection of full genomes of cetacean morbilliviruses, which would allow a deeper understanding of their evolution, as recently assessed for ruminant morbilliviruses^23^. Moreover, NGS coupled with advanced phylogenetics and phylogeography would permit to reconstruct the spatio-temporal spread of cetacean morbilliviruses, to trace the transmission routes between cetacean species and to identify possible reservoirs responsible for viral persistence and reoccurrence.

## Methods

### Material

The striped dolphin was an adult female stranded along the Ligurian coast (north-western Italy) in October 2008. At the time of stranding the animal underwent necropsy^24^. The striped dolphin appeared emaciated. Briefly, *post-mortem* investigations revealed brain congestion, granulomatous myocarditis, lymphoid depletion of lymph nodes and spleen, and chronic interstitial nephritis. Mild non-suppurative meningo-encephalitis and moderate interstitial broncopneumonia with syncytial cells, suggestive of morbillivirus infection, were also observed. DMV infection was detected by IHC and RT-PCR^25^ in CNS, lungs and lymph nodes. A *Toxoplasma gondii* infection was also detected through PCR^26^ and IHC^27^ in CNS, lungs and heart and by serology (IFAT)^15^.

### Viral isolation

In 2015, an archive frozen brain sample of the striped dolphin was processed for virus isolation using Vero.DogSLAMtag cell line (kindly provided by Dr. Yusuke Yanagi, Faculty of Medical Science, Kyushu University, Japan).Brain tissue was submitted to standard virological procedures and, in the first instance, viral isolation was attempted on Vero (ATCC® CCL-81™) and Vero h/SLAM (Sigma Aldrich 04091501) cell lines, but it was unsuccessful. Briefly, sample was ground and homogenized in Dulbecco’s Modified Eagle’s Medium (Sigma Aldrich) containing higher antibiotic concentrations (5X), clarified by centrifugation (3500× rcf for 15 min at 4 °C), filtered through disposable filters with an average pore diameter of 0.45 μm and 100 µL inoculated onto subconfluent monolayer Vero.DogSLAMtag cell cultures. To allow viral adsorption, plates were incubated for 60 min. at 37 ± 1 °C with 5% CO_2_ with gentle rocking every 15 min. One ml of MEM (Sigma Aldrich) was dispensed in each well and plates were subsequently incubated at 37 ± 1 °C with 5% CO_2_ for a maximum of 5–7 days. Cultures were daily inspected under a microscope for the occurrence of cytopathic effect (CPE). Re-inoculation of wells showing at least 80% of CPE or blind passage from wells not exhibiting CPE was carried out until an overall of three serial passage were achieved. For re-inoculation, samples showing or not CPE were harvested by freezing-thawing, and sub-cultured onto fresh cells and incubated for further 5–7 days in the same conditions as previously described.

Only wells with CPE were rinsed three times in Phosphate-buffered saline (PBS, pH 7.4) and fixed in C_2_H_6_O for 2 h at 4 °C. Giemsa, filtered with a 0,45 µm pore size membrane and diluted 1/10 in PBS, was used to stain the cell monolayers (1 h at room temperature).

### RNA extraction and NGS analysis

Cells were harvested by freezing-thawing and the cell pellet was dissolved in 0.8 ml of TRI Reagent (Sigma-Aldrich). RNA isolation was carried out using the standard TRI Reagent protocol. RNA was DNase treated (Baseline-Zero^TM^ DNase, Zymo Research), concentrated with the RNA Clean & Concentrator^TM^ kit (Zymo Research), and quantified using the Qubit RNA HS Assay kit on the Qubit 3.0 Fluorometer (Thermo Fisher Scientific). Total RNA concentration was 48 ng/µL. Five µL of total RNA were reverse transcribed to ss-cDNA employing the Reverse Transcription System kit (Promega) in presence of 50 ng of random hexamers. After purification with magnetic beads (AMPure XP, Ambion), ds-cDNA was synthesized using the NEBNext® mRNA Second Strand Synthesis Module (New England Biolabs), and again purified with magnetic beads. Both RNA and ds-cDNA were tested by real time PCR to assess semi-quantitatively the viral titer; an assay based on the QuantiFast® SYBR® Green RT-PCR kit (Qiagen) was applied for this aim, employing CeMV-He 1 (5′-CRTTGATACTYGTGGGTGTG-3′) and CeMV-He 2 (5′-TGTTAACTTCTGGGGCATCC-3′) primers^28^.

Ds-cDNA was quantified using the Qubit dsDNA HS Assay kit on the Qubit 3.0 Fluorometer (Thermo Fisher Scientific), and properly diluted to 0.2 ng/µL for downstream application of the Nextera XT DNA Library Prep Kit protocol (Illumina). Library preparation was carried out using 1 ng of input ds-cDNA according to the manufacturer’s recommendations and went through the following steps: DNA random fragmentation by tagmentation (150–1,000 bp fragments); ligation with Illumina universal adapters; PCR amplification and insertion of sample-specific index; library normalization.

Libraries were submitted to massive parallel sequencing on a MiSeq platform (Illumina) in paired-end mode (2 × 300 bp) loading a MiSeq Reagent Kit v3 cartridge (Illumina).

### Sanger sequencing

Total RNA was isolated from the brain of the striped dolphin (TRI Reagent, Sigma-Aldrich). Five μl of RNA were reverse transcribed by random primers and the High Capacity Reverse Transcription kit (Life Technologies). Primers flanking the coding mutations detected by NGS were designed using Primer3 (http://frodo.wi.mit.edu/cgi-bin/primer3/) (Table S1). PCR was carried out using a FastStart Taq DNA polymerase kit (Roche) in a final volume of 50 µl containing 5 µl PCR buffer (10X), 1 µl dNTPs (10 mM), 1.5 µL of each primer (10 µM), 0.2 µl Taq (5 U/µl), and 1.5 µl cDNA. The following thermal conditions were applied: activation of Taq polymerase at 95 °C for 10 min, followed by 40 cycles of denaturation at 94 °C for 1 min; annealing at 60 °C for 1 min; extension at 72 °C for 2 min; and final elongation at 72 °C for 7 min. Amplicons were purified and sequenced on both strands using a BigDye Terminator v3.1 cycle sequencing kit (Life Technologies) on a 3130 Genetic Analyzer (Life Technologies). Manual editing was performed with Sequencing Analysis software, version 5.2 (Life Technologies). The sequences were aligned to the DMV_IZSPLV_2008 genome using the SeqMan software (Lasergene).

### Bioinformatics analyses

Reads were quality checked using FastQC v. 0.11.3 (http://www.bioinformatics.bbsrc.ac.uk/projects/fastqc/), and quality and adapter trimming was then performed using the script bbduk2.sh, included in the BBMap v. 35.68 package (https://sourceforge.net/projects/bbmap/). Reads passing the quality filter were aligned to the DMV reference genome (GenBank Acc. no. NC_005283) using the Burrows*-*Wheeler algorithm implemented in BWA v. 0.7.12 software. Reads were also assembled *de novo* using two different software: the de novo assembler provided in CLC Genomics Workbench 10.1.1 (Qiagen) and the SPAdes assembler version 3.10.0. In CLC, default de novo parameters (bubble size 98; k-mer size 45) were applied. Reads were mapped back to contigs with the following mapping options: match score 1, mismatch cost 2, insertion and deletion cost 3, similarity fraction 0.8 and length fraction 0.5. Alignment mode was set to local and match mode to random, minimum contig length was 200 bp. De novo assembly in SPAdes was performed using the default settings for paired-end read, with automatic iterative k-mer length determination (21, 33, 55, 77, 99, 127); the contig of interest was used as reference for mapping with BWA and then SAMtools v.0.1.19 was used to retrieve information about the coverage^29^.

Read mapping was visualized with Tablet v. 1.15.09.01 software and a genome consensus was obtained. The newly generated DMV genome was deposited in GenBank under Acc. no. MF589987.

### Phylogenetic analysis

Multiple sequence alignment was performed with BioEdit version 7.0.5.2 using CLUSTAL W. Sequence similarities were calculated using MegAlign (Lasergene package, DNASTAR Inc.). The nucleotide p-distance was computed with the *dist.dna* function (model = “raw”) implemented in the *ape* package within the R statistical environment^30^. The obtained values were plotted on a barplot using the *ggplot2* package. Nucleotide substitution models were evaluated using jModelTest2^31^ and the best model was selected according to the AIC analysis. MEGA7 was used for phylogeny inference according to the maximum likelihood (ML) criterion^32^. The nucleotide substitution model was set according to jModelTest2 output and was General Time Reversible with Invariant sites (GTR + I). The robustness of the hypothesis was tested in 1,000 non-parametric bootstrap iterations.

### Data availability

All data generated during and/or analysed during the current study are available from the corresponding author on reasonable request.

## Electronic supplementary material

Supplementary Information
