# Supplementary material for: Efficient isolation on Vero.DogSLAMtag cells and full genome characterization of Dolphin Morbillivirus (DMV) by next generation sequencing
Source: Sci Rep. 2018 Jan 16;8:860. doi: 10.1038/s41598-018-19269-2 (PMC5770449; doi:10.1038/s41598-018-19269-2)
Supplement: Supplementary file 1 — Supplementary Information [file 41598_2018_19269_MOESM1_ESM.pdf]

Supplementary information

Efficient isolation on Vero.DogSLAMtag cells and full genome characterization of Dolphin Morbillivirus (DMV) by next generation sequencing.

Simone Peletto[1,]*, Claudio Caruso[1], Francesco Cerutti[1], Paola Modesto[1], Cristina Biolatti[1], Alessandra Pautasso[1], Carla Grattarola[1], Federica Giorda[1], Sandro Mazzariol[2], Walter Mignone[1], Loretta Masoero[1], Cristina Casalone[1], Pier Luigi Acutis[1]

[1] *Istituto Zooprofilattico Sperimentale del Piemonte, Liguria e Valle d'Aosta, Turin, Italy*

[2] *Department of Comparative Biomedicine and Food Science, University of Padua, Padua, Italy*

Supplementary Figure S1. Nucleoprotein (N) sequences of the DMV genomes representative of the 1990-'92 and 2006-'08 outbreaks in the Mediterranean Sea. Points indicate identical residues. Host species (Sc = *Stenella coeruleoalba*; Gme = *Globicefala melas*), collection date and accession numbers are indicated for each sequence. The sequence marked by asterisk (*) has been newly determined in this study.

```
                                    10        20        30        40        50        60        70        80        90       100
                            ....|....|....|....|....|....|....|....|....|....|....|....|....|....|....|....|....|....|....|....|
 DMV_Sc/1990/Me_NC_005283   MATLLRSLALFKRNKDRTPLIAGSGGAIRGIKHVIVVPVPGDSSIVTRSRLLDRLVRLAGDPYISGPKLTGVMISILSLFVESPSQLIQRITDDPDVSIR
*DMV_IZSPLV_Sc/2008/Me      ....................T.....................................................V..........................
 DMV_Gme/2007/Me_HQ829972   ....................T...............................................................................
 DMV_Sc/2007/Me_HQ829973    ....................T...............................................................................

                                   110       120       130       140       150       160       170       180       190       200
                            ....|....|....|....|....|....|....|....|....|....|....|....|....|....|....|....|....|....|....|....|
 DMV_Sc/1990/Me_NC_005283   LVEVIQSEKSLSGLTFASRGANMEDEADDYFSIQAGEEGDTRGTHWFENKEIVEIEVQDPEEFNILLASILAQIWILLAKAVTAPDTAADSETRRWIKYT
*DMV_IZSPLV_Sc/2008/Me      ....................................................................................................
 DMV_Gme/2007/Me_HQ829972   ....................................................................................................
 DMV_Sc/2007/Me_HQ829973    ....................................................................................................

                                   210       220       230       240       250       260       270       280       290       300
                            ....|....|....|....|....|....|....|....|....|....|....|....|....|....|....|....|....|....|....|....|
 DMV_Sc/1990/Me_NC_005283   QQRRVVGEFRLDKGWLDAVRNRIAEDLSLRRFMVALILDIKRTPGNKPRIAEMICDIDTYIVEAGLASFILTIKFGIETMYPALGLHEFSGELTTVESLM
*DMV_IZSPLV_Sc/2008/Me      ....................................................................................................
 DMV_Gme/2007/Me_HQ829972   ....................................................................................................
 DMV_Sc/2007/Me_HQ829973    ....................................................................................................

                                   310       320       330       340       350       360       370       380       390       400
                            ....|....|....|....|....|....|....|....|....|....|....|....|....|....|....|....|....|....|....|....|
 DMV_Sc/1990/Me_NC_005283   NLYQQMGETAPYMVILENSIQNKFSAGSYPLLWSYAMGVGVELENSMGGLNFGRSYFDPAYFRLGQEMVRRSAGKVSSSLAAELGITAEDAKLVSEIAAQ
*DMV_IZSPLV_Sc/2008/Me      ....................................................................................................
 DMV_Gme/2007/Me_HQ829972   ....................................................................................................
 DMV_Sc/2007/Me_HQ829973    ....................................................................................................

                                   410       420       430       440       450       460       470       480       490       500
                            ....|....|....|....|....|....|....|....|....|....|....|....|....|....|....|....|....|....|....|....|
 DMV_Sc/1990/Me_NC_005283   ANDDRANRAIGPKQNQISFLHPDRGDASTPGNILRANEGDGSTRMKRGGNIATPKGTSIDQTSTTLSKDTLDIDEQSDNTDDPISIQKSAEALAKMRAMA
*DMV_IZSPLV_Sc/2008/Me      ...................................T.........................A......................................
 DMV_Gme/2007/Me_HQ829972   ...................................T.........................A......................................
 DMV_Sc/2007/Me_HQ829973    ...................................T.................L.......A......................................

                                   510       520
                            ....|....|....|....|....
 DMV_Sc/1990/Me_NC_005283   KLLENQGPRDVTAHVYNDKDLLG*
*DMV_IZSPLV_Sc/2008/Me      .......................*
 DMV_Gme/2007/Me_HQ829972   .......................*
 DMV_Sc/2007/Me_HQ829973    .......................*
```

Supplementary Figure S2. Phosphoprotein (P) sequences of the DMV genomes representative of the 1990-'92 and 2006-'08 outbreaks in the Mediterranean Sea. Points indicate identical residues. Host species (Sc = *Stenella coeruleoalba*; Gme = *Globicefala melas*), collection date and accession numbers are indicated for each sequence. The sequence marked by asterisk (*) has been newly determined in this study.

```
                                 10        20        30        40        50        60        70        80        90       100
                         ....|....|....|....|....|....|....|....|....|....|....|....|....|....|....|....|....|....|....|....|
 DMV_Sc/1990/Me_NC_005283 MAEEQAYHVNKGLECLKSLRENPPDAVEIKEAQIIRSKAACEESSESHHQDNSEKDTLDFDESCSSAIRPETYRMLLGDDTGFRAPGYIPNEGEPEPGDI
*DMV_IZSPLV_Sc/2008/Me    ....................................................................................................
 DMV_Gme/2007/Me_HQ829972 ....................................................................................................
 DMV_Sc/2007/Me_HQ829973  ....................................................................................................

                                110       120       130       140       150       160       170       180       190       200
                         ....|....|....|....|....|....|....|....|....|....|....|....|....|....|....|....|....|....|....|....|
 DMV_Sc/1990/Me_NC_005283 GKEEPAVRCYHVYDHGGQAVEGVKDADLLVVPTGSDDDAEFRDGDESSLESDGESGTVDTRGNSSSNRGSAPRIKVERSSDVETISSEELQGLIRSQSQK
*DMV_IZSPLV_Sc/2008/Me    ..................E...........................................S.....................................
 DMV_Gme/2007/Me_HQ829972 ..................E...........................................S.....................................
 DMV_Sc/2007/Me_HQ829973  ..................E...........................................S.....................................

                                210       220       230       240       250       260       270       280       290       300
                         ....|....|....|....|....|....|....|....|....|....|....|....|....|....|....|....|....|....|....|....|
 DMV_Sc/1990/Me_NC_005283 HNGFGVDRFLKVPPIPTSVPLDPAPKSIKKGTGERSALSGTETEFSLTGGATRLAQESRWASSESSAPAENVRQSVTNAERTQKPPQGSGTTASQKSQNN
*DMV_IZSPLV_Sc/2008/Me    ....................................................................................................
 DMV_Gme/2007/Me_HQ829972 ....................................................................................................
 DMV_Sc/2007/Me_HQ829973  ....................................................................................................

                                310       320       330       340       350       360       370       380       390       400
                         ....|....|....|....|....|....|....|....|....|....|....|....|....|....|....|....|....|....|....|....|
 DMV_Sc/1990/Me_NC_005283 GHSDDEYEDELFMEVQEIKTAITKINEDNQQIISKLDSIMLLKGEIESIKKQINKQNITISTIEGHLSSIMIAIPGFGKDPNDPTADVELNPDLRPIISR
*DMV_IZSPLV_Sc/2008/Me    ............L.......................................................................................
 DMV_Gme/2007/Me_HQ829972 ............L.......................................................................................
 DMV_Sc/2007/Me_HQ829973  ............L.......................................................................................

                                410       420       430       440       450       460       470       480       490       500
                         ....|....|....|....|....|....|....|....|....|....|....|....|....|....|....|....|....|....|....|....|
 DMV_Sc/1990/Me_NC_005283 DAGRALAEVLKRPAVERNPKVTPKVHPGSKGQILRDLQLKPVDRKMSSAVGFVPTDDLPSRSVLRSMIKSSNLESEHKRSMIGLLNDVKSGKDLGEFYQM
*DMV_IZSPLV_Sc/2008/Me    ....................................................................................................
 DMV_Gme/2007/Me_HQ829972 ....................................................................................................
 DMV_Sc/2007/Me_HQ829973  ....................................................................................................

                         ....|..
 DMV_Sc/1990/Me_NC_005283 VKKIIK*
*DMV_IZSPLV_Sc/2008/Me    ......*
 DMV_Gme/2007/Me_HQ829972 ......*
 DMV_Sc/2007/Me_HQ829973  ......*
```

Supplementary Figure S3. Matrix (M) protein sequences of the DMV genomes representative of the 1990-'92 and 2006-'08 outbreaks in the Mediterranean Sea. Points indicate identical residues. Host species (Sc = *Stenella coeruleoalba*; Gme = *Globicefala melas*), collection date and accession numbers are indicated for each sequence. The sequence marked by asterisk (*) has been newly determined in this study.

```
                                    10        20        30        40        50        60        70        80        90       100
                           ....|....|....|....|....|....|....|....|....|....|....|....|....|....|....|....|....|....|....|....|
 DMV_Sc/1990/Me_NC_005283  MTEVYDFDRSAWDVKGSIAPIEPTTYPDGRLIPQVRVIDPGLGDRKDECFMYIFLLGILEDNDIMSPPIGRTFGSLPLGVGRSTAKPEELLKEATELDIV
*DMV_IZSPLV_Sc/2008/Me     ..........................................................V.........................................
 DMV_Gme/2007/Me_HQ829972  ..........................................................V.........................................
 DMV_Sc/2007/Me_HQ829973   ..........................................................V.........................................

                                   110       120       130       140       150       160       170       180       190       200
                           ....|....|....|....|....|....|....|....|....|....|....|....|....|....|....|....|....|....|....|....|
 DMV_Sc/1990/Me_NC_005283  VRRTAGLNEKLVFYNNTPLMLLTPWKKVLTAGSVFSANQVCNAVNLIPLDTPQRFRVVYMSITRLSDNGCYRVPRKMLEFRSANALAFNILVTIRIENAG
*DMV_IZSPLV_Sc/2008/Me     ....................................................................................................
 DMV_Gme/2007/Me_HQ829972  ....................................................................................................
 DMV_Sc/2007/Me_HQ829973   ....................................................................................................

                                   210       220       230       240       250       260       270       280       290       300
                           ....|....|....|....|....|....|....|....|....|....|....|....|....|....|....|....|....|....|....|....|
 DMV_Sc/1990/Me_NC_005283  IVSRPYMSMMRDPQATFMIHIGNFRRKKNEAYSADYCKMKIEKMGLVFALGGIGGTSLHIRCTGKMSKTLHAQLGFKKILCYPLMDVNEDLNRYLWRAEC
*DMV_IZSPLV_Sc/2008/Me     ....................................................................................................
 DMV_Gme/2007/Me_HQ829972  ....................................................................................................
 DMV_Sc/2007/Me_HQ829973   ....................................................................................................

                                   310       320       330
                           ....|....|....|....|....|....|....|.
 DMV_Sc/1990/Me_NC_005283  KIVRIQAVLQPSVPQEFRVYDDVIINDDQGLFKIL*
*DMV_IZSPLV_Sc/2008/Me     ...................................*
 DMV_Gme/2007/Me_HQ829972  ...................................*
 DMV_Sc/2007/Me_HQ829973   ...................................*
```

Supplementary Figure S4. Fusion (F) protein sequences of the DMV genomes representative of the 1990-'92 and 2006-'08 outbreaks in the Mediterranean Sea. Points indicate identical residues. Host species (Sc = *Stenella coeruleoalba*; Gme = *Globicefala melas*), collection date and accession numbers are indicated for each sequence. The sequence marked by asterisk (*) has been newly determined in this study.

```
                                   10        20        30        40        50        60        70        80        90       100
                           ....|....|....|....|....|....|....|....|....|....|....|....|....|....|....|....|....|....|....|....|
 DMV_Sc/1990/Me_NC_005283  MAASNGGVMYQSFLTIIILVIMTEGQIHWGNLSKIGIVGTGSASYKVMTRPNHQYLVIKLMPNVTMIDNCTRTEVTEYRKLLKTVLEPVKNALTVITKNI
*DMV_IZSPLV_Sc/2008/Me     .V.................................................S................................................
 DMV_Gme/2007/Me_HQ829972  .V.................................................S................................................
 DMV_Sc/2007/Me_HQ829973   ...................................................S................................................

                                  110       120       130       140       150       160       170       180       190       200
                           ....|....|....|....|....|....|....|....|....|....|....|....|....|....|....|....|....|....|....|....|
 DMV_Sc/1990/Me_NC_005283  KPIQSLTTSRRSKRFAGVVLAGVALGVATAAQITAGVALHQSIMNSQSIDNLRTSLEKSNQAIEEIRQASQETVLAVQGVQDFINNELIPSMHQLSCEML
*DMV_IZSPLV_Sc/2008/Me     .....................................................A..............................................
 DMV_Gme/2007/Me_HQ829972  .....................................................A..............................................
 DMV_Sc/2007/Me_HQ829973   ..................................................S..A..............................................

                                  210       220       230       240       250       260       270       280       290       300
                           ....|....|....|....|....|....|....|....|....|....|....|....|....|....|....|....|....|....|....|....|
 DMV_Sc/1990/Me_NC_005283  GQKLGLKLLRYYTEILSIFGPSLRDPVSAEISIQALSYALGGDINKILEKLGYSGADLLAILESRGIKAKVTHVDLEGYFIVLSIAYPTLSEVKGVIVHK
*DMV_IZSPLV_Sc/2008/Me     ....................................................................................................
 DMV_Gme/2007/Me_HQ829972  ....................................................................................................
 DMV_Sc/2007/Me_HQ829973   ....................................................................................................

                                  310       320       330       340       350       360       370       380       390       400
                           ....|....|....|....|....|....|....|....|....|....|....|....|....|....|....|....|....|....|....|....|
 DMV_Sc/1990/Me_NC_005283  LEAVSYNLGSQEWYTTLPKYVATNGYLISNFDESSCAFMSEVTICSQNALYPMSPLLQQCLRGSTASCARSLVSGTIGNRFILSKGNLIANCASVLCKCY
*DMV_IZSPLV_Sc/2008/Me     ....................................................................................................
 DMV_Gme/2007/Me_HQ829972  ....................................................................................................
 DMV_Sc/2007/Me_HQ829973   ....................................................................................................

                                  410       420       430       440       450       460       470       480       490       500
                           ....|....|....|....|....|....|....|....|....|....|....|....|....|....|....|....|....|....|....|....|
 DMV_Sc/1990/Me_NC_005283  STGTIISQDPDKLLTFVAADKCPLVEVDGITIQVGSREYPDSVYVSRIDLGPAISLEKLDVGTNLGSALTKLDNAKDLLDSSNQILENVRRSSFGGAMYI
*DMV_IZSPLV_Sc/2008/Me     ....................................................................................................
 DMV_Gme/2007/Me_HQ829972  ....................................................................................................
 DMV_Sc/2007/Me_HQ829973   ....................................................................................................

                                  510       520       530       540       550
                           ....|....|....|....|....|....|....|....|....|....|...
 DMV_Sc/1990/Me_NC_005283  GILVCAGALVILCVLVYCCRRHCRKRVQTPPKATPGLKPDLTGTTKSYVRSL*
*DMV_IZSPLV_Sc/2008/Me     ....................................................*
 DMV_Gme/2007/Me_HQ829972  ....................................................*
 DMV_Sc/2007/Me_HQ829973   ....................................................*
```

Supplementary Figure S5. Hemagglutinin (H) protein sequences of the DMV genomes representative of the 1990-'92 and 2006-'08 outbreaks in the Mediterranean Sea. Points indicate identical residues. Host species (Sc = *Stenella coeruleoalba*; Gme = *Globicefala melas*), collection date and accession numbers are indicated for each sequence. The sequence marked by asterisk (*) has been newly determined in this study.

```
                                  10        20        30        40        50        60        70        80        90       100
                          ....|....|....|....|....|....|....|....|....|....|....|....|....|....|....|....|....|....|....|....|
 DMV_Sc/1990/Me_NC_005283 MSSPRDKVDAFYKDIPRPRNNRVLLDNERVIIERPLILVGVLAVMFLSLVGLLAIAGVRLQKATTNSIEVNRKLSTNLETTVSIEHHVKDVLTPLFKIIG
*DMV_IZSPLV_Sc/2008/Me    ....................................................................................................
 DMV_Gme/2007/Me_HQ829972 ....................................................................................................
 DMV_Sc/2007/Me_HQ829973  ....................................................................................................

                                 110       120       130       140       150       160       170       180       190       200
                          ....|....|....|....|....|....|....|....|....|....|....|....|....|....|....|....|....|....|....|....|
 DMV_Sc/1990/Me_NC_005283 DEVGLRMPQKLTEIMQFISNKIKFLNPDREYDFNDLHWCVNPPDQVKIDYAQYCNHIAAEELIVTKFKELMNHSLDMSKGRIFPPKNCSGSVITRGQTIK
*DMV_IZSPLV_Sc/2008/Me    ...............................................................A....................................
 DMV_Gme/2007/Me_HQ829972 ...............................................................A....................................
 DMV_Sc/2007/Me_HQ829973  ...............................................................A....................................

                                 210       220       230       240       250       260       270       280       290       300
                          ....|....|....|....|....|....|....|....|....|....|....|....|....|....|....|....|....|....|....|....|
 DMV_Sc/1990/Me_NC_005283 PGLTLVNIYTTRNFEVSFMVTVISGGMYGKTYFLKPPEPDDPFEFQAFRIFEVGLVRDVGSREPVLQMTNFMVIDEDEGLNFCLLSVGELRLAAVCVRGR
*DMV_IZSPLV_Sc/2008/Me    ....................................................................................................
 DMV_Gme/2007/Me_HQ829972 ....................................................................................................
 DMV_Sc/2007/Me_HQ829973  ....................................................................................................

                                 310       320       330       340       350       360       370       380       390       400
                          ....|....|....|....|....|....|....|....|....|....|....|....|....|....|....|....|....|....|....|....|
 DMV_Sc/1990/Me_NC_005283 PVVTKDIGGYKDEPFKVVTLGIIGGGLSNQKTEIYPTIDSSIEKLYITSHRGIIRNSKARWSVPAIRSDDKDKMEKCTQALCKSRPPPSCNSSDWEPLTS
*DMV_IZSPLV_Sc/2008/Me    ....................................................................................................
 DMV_Gme/2007/Me_HQ829972 ....................................................................................................
 DMV_Sc/2007/Me_HQ829973  ....................................................................................................

                                 410       420       430       440       450       460       470       480       490       500
                          ....|....|....|....|....|....|....|....|....|....|....|....|....|....|....|....|....|....|....|....|
 DMV_Sc/1990/Me_NC_005283 NRIPAYAYIALEIKEDSGLELDITSNYGPLIIHGAGMDIYEGPSSNQDWLAIPPLSQSVLGVINKVDFTAGFDIKPHTLTTAVDYESGKCYVPVELSGAK
*DMV_IZSPLV_Sc/2008/Me    ....................................................................................................
 DMV_Gme/2007/Me_HQ829972 ....................................................................................................
 DMV_Sc/2007/Me_HQ829973  ....................................................................................................

                                 510       520       530       540       550       560       570       580       590       600
                          ....|....|....|....|....|....|....|....|....|....|....|....|....|....|....|....|....|....|....|....|
 DMV_Sc/1990/Me_NC_005283 DQDLKLESNLVVLPTKDFGYVTATYDTSRSEHAIVYYVYDTARSSSYFFPFRIKARGEPIYLRIECFPWSRQLWCHHYCMINSTVSNEIVVVDNLVSINM
*DMV_IZSPLV_Sc/2008/Me    ....................................................................................................
 DMV_Gme/2007/Me_HQ829972 ....................................................................................................
 DMV_Sc/2007/Me_HQ829973  ....................................................................................................


                          ....|
 DMV_Sc/1990/Me_NC_005283 SCSR*
*DMV_IZSPLV_Sc/2008/Me    ....*
 DMV_Gme/2007/Me_HQ829972 ....*
 DMV_Sc/2007/Me_HQ829973  ....*
```

Supplementary Table S1. Primers used for PCR amplification and Sanger sequencing to confirm coding nucleotide variants of the DMV_IZSPLV_2008 strain.

Primer	Sequence (5’ – 3’)	*nt* position (NC_005283.1)	Amplicon size (bp)
DMV_F1	GAGACTGCACCGTACATGG	1029-1047	800
DMV_R1	TGACATGATAGGCCTGCTCC	1809-1828	
DMV_F2	GAGCACCAGGTTACATCCCT	2051-2070	280
DMV_R2	TCGACCTTAATCCTGGGAGC	2311-2330	
DMV_F3	GCTCTGCTGTGGGATTTGTC	3140-3159	580
DMV_R3	TTCGGTGGCCTCTTTAAGCA	3700-3719	
DMV_F4	TACTTGTGGCGTGCAGAATG	4311-4330	901
DMV_R4	CTCCTAGCGACAGTGACTCA	5192-5211	
DMV_F5	TCCGTGGTCAAATATTGTCGA	5216-5236	1000
DMV_R5	CCACTCTTGAGATCCTAAGTTGT	6193-6215	
DMV_F6	TGGCACCATAATTAGCCAGGA	6482-6502	1191
DMV_R6	GGTGATGACTGAGCCCGAG	7654-7672	
DMV_F7	AACCACCCGAACCTGATGAT	7794-7813	458
DMV_R7	GGGTGGTCTACTCTTGCACA	8232-8251	
DMV_F8	TTTGGGAGCCTACATGCAGA	9494-9513	660
DMV_R8	CTCACATTGTTGGCTGCTGT	10134-10153	
DMV_F9	TGTAACAGCAGCCAACAATGT	10130-10150	1014
DMV_R9	TCCAACCTCTTGTGTAGCCA	11124-11143	
DMV_F10	TCCATGACGAAAGCCAAGAG	12142-12161	1064
DMV_R10	TCTAGGTGCAGTACGGTGAC	13186-13205	
DMV_F11	AGGAGATTTTGGGACTGTGGA	13659-13679	430
DMV_R11	TGATCTGTTTAACCGCCCCT	14069-14088	
